# Prognostic Role of Tumor Budding in Carcinoma Tongue: A Systemic Review and Meta-Analysis

**DOI:** 10.7759/cureus.9316

**Published:** 2020-07-21

**Authors:** Uday Karjol, Pavan Jonnada, Vinitha Annavarjula, Sushma Cherukuru, Ajay Chandranath, Ali Anwar

**Affiliations:** 1 Surgical Oncology, Kidwai Memorial Institute of Oncology, Bangalore, IND; 2 Oral and Maxillofacial Surgery, Surgical Oncology, Kidwai Memorial Institute of Oncology, Bangalore, IND; 3 Pathology, AmPath Laboratories, Hyderabad, IND

**Keywords:** oral cancer, tumor budding, tongue cancer, survival, lymph node metastasis

## Abstract

Introduction

Tumor budding is defined as a cluster of cells that invade the stroma. This has recently been studied to be associated with lymph node metastasis (LNM) and poor overall survival (OS) rate. The reliability and reproducibility of this histopathological feature make it a valid prognostic indicator in tongue carcinomas, which often have an unpredictable prognosis. The objective of this study was to group the studies that elucidate the prognostic role of tumor budding in tongue cancers.

Methods

A systematic database search was performed in MEDLINE, Embase, and Google Scholar for relevant studies that reported tumor budding in tongue cancer. The relevant articles were independently screened by two authors for selection and data extraction. As a result, a list of such studies, clinical trials, and references, published in English up to March 2020, was obtained, and a total of 1448 patients in nine studies were included in this meta-analysis. Statistical analysis was conducted using RevMan software 5.3 (The Nordic Cochrane Centre, Cochrane Collaboration, Copenhagen).

Results

A higher tumor budding score was significantly correlated with LNM (hazard ratio (HR): 3.07; 95% confidence interval (CI): 2.08-4.52; p≤.00001) and poor OS (HR: 2.40; 95% CI: 1.84-3.14; p≤.00001) in tongue cancer.

Conclusions

Our present study demonstrates that tumor budding is an independent predictor of LNM and OS in tongue cancer. Tumor budding should be considered a parameter in future oncological staging systems.

## Introduction

Oral cavity cancer is one of the most common malignancies worldwide, with high frequency in southern Asia as well as the Pacific Islands, and accounts for 30% of all cancers in these regions [1]. Cancers involving the lips, mouth, and tongue define oral cancer. This definition is adopted from the International Classification of Diseases coding scheme, the World Health Organization case definition, and the International Agency for Research and Cancer [2].

Among the oral cancers, tongue carcinomas are one of the most common cancers with unpredictable lymphatic spread and distant metastasis. The prognostication of tongue carcinomas is usually done by traditional biomarkers and histopathological features. The pathological features, such as perineural invasion and lymphovascular invasion, have shown to have prognostic value but do not have reliable prognostic significance [3].

Recently, tumor budding has been demonstrated as a prognostic marker and is a characteristic of the aggressiveness of cancer [4]. Tumor budding is characterized by pathological alveoli harboring one to four cancer cells at the invasive front [5]. Tumor budding is a dependable prognostic marker of oral cavity cancers. However, it is not considered a part of the standard staging of these cancers.

This study aims at conducing that tumor-budding pattern is a reliable prognostic factor and its role in determining the risk of lymph node metastasis (LNM) and overall survival (OS) of the patients with carcinoma tongue.

## Materials and methods

Search strategy

We performed a systematic literature search on the MEDLINE, Embase, and Google Scholar databases for articles published before March 2020 using the following strategy. The articles were searched using Medical Education Subject Headings (MeSH) keywords "(oral cancer) OR (tongue cancer)) OR (tongue carcinoma)) AND (tumor-budding)) AND (lymph node)) AND (metastasis)." Preferred Reporting Items for Systematic Reviews and Meta-Analyses (PRISMA) guidelines were followed to search and report the articles.

Study selection

All studies that reported an association of tumor budding with LNM and OS for tongue cancer patients were identified by a comprehensive computer-based search. Two authors (PJ and VA) independently assessed titles and abstracts for eligibility. The reference lists were scanned for similar articles. All the screened articles were assessed to match the eligibility and any discrepancy was resolved through discussion. The studies that met the following inclusion criteria were included in the meta-analysis. The criteria of inclusion were articles published in English that were clinical trials and studies that compared the OS and LNM on tumor budding. Studies containing republished data, inability to extract data from the published results, and publications in the form of editorials, comments, review articles, meeting abstracts, or those that omitted reported outcomes were excluded.

Data extraction

The relevant data were extracted from the screened articles independently by two authors (PJ and VA). The data extracted from the articles that fulfilled the criteria were the following: the first author and the basic characteristics of the study, year of publication, study setting, design of the study, duration of the study, data sources, multivariate adjustments; the basic patient characteristics, including age, gender, stage, treatment, and survival periods; comparative outcomes, including hazard ratio (HR) for LNM, and OS on different tumor-budding subgroups.

Quality assessment

Two authors (PJ and VA) independently appraised the quality of each included study using the Oxford Quality Scoring System [6]. The included studies are shown in Table 1. If the study did not meet more than one criterion in the selection domain or if the compatibility domain did not have a score and if more than one criterion in the outcome domain were not met, it was considered to be of poor quality and hence excluded. Any disagreements between reviewers were resolved by consensus.

**Table 1:** Characteristics of included studies RCS: retrospective cohort study; LNM: lymph node metastasis; OS: overall survival; OQSS: Oxford Quality Scoring System

Author name	Year	Study design	Sample size	Endpoint	OQSS	Tumour-budding cut-off
Wang et al. [4]	2011	RCS	230	OS	3	<5, ≥5
Nanxie et al. [7]	2014	RCS	195	LNM, OS	3	<5, ≥5
Angadi et al. [8]	2015	RCS	75	LNM	4	<10, ≥10
Hori et al. [9]	2017	RCS	48	LNM	4	<5, ≥5
Yamakawa et al. [10]	2018	RCS	337	LNM	3	<5, ≥5
Shimizu et al. [11]	2018	RCS	91	LNM, OS	2	<5, ≥5
Sakata et al. [12]	2018	RCS	97	LNM	3	<4,≥4
Ebihara et al. [5]	2019	RCS	64	LNM, OS	4	<5, ≥5
Elseragy et al. [3]	2019	RCS	311	OS	3	<5, ≥5

Statistical analysis

RevMan software version 5.3 (The Nordic Cochrane Centre, Cochrane Collaboration, Copenhagen) was used for statistical analysis. HR was analyzed as a continuous variable and 95% CI was recorded. Heterogeneity was assessed using the χ^2^ and I^2^ tests. I^2^ of 0-30, 30-60, 60-70, and >75% represent low, moderate, substantial, and considerable heterogeneity, respectively. Studies with a P-value of <.1 and I^2^ > 40% indicated substantial heterogeneity. The fixed-effects model was used with P >.10 and I^2^<25%. If significant heterogeneity existed in the fixed-effects model then the random-effects model was used to estimate the pooled HR. The Z-test was used to determine the pooled HR, and the significance was set to reject the null hypothesis at P <.05. Funnel plots were adapted to investigate possible bias.

## Results

Studies included

**Figure 1:**
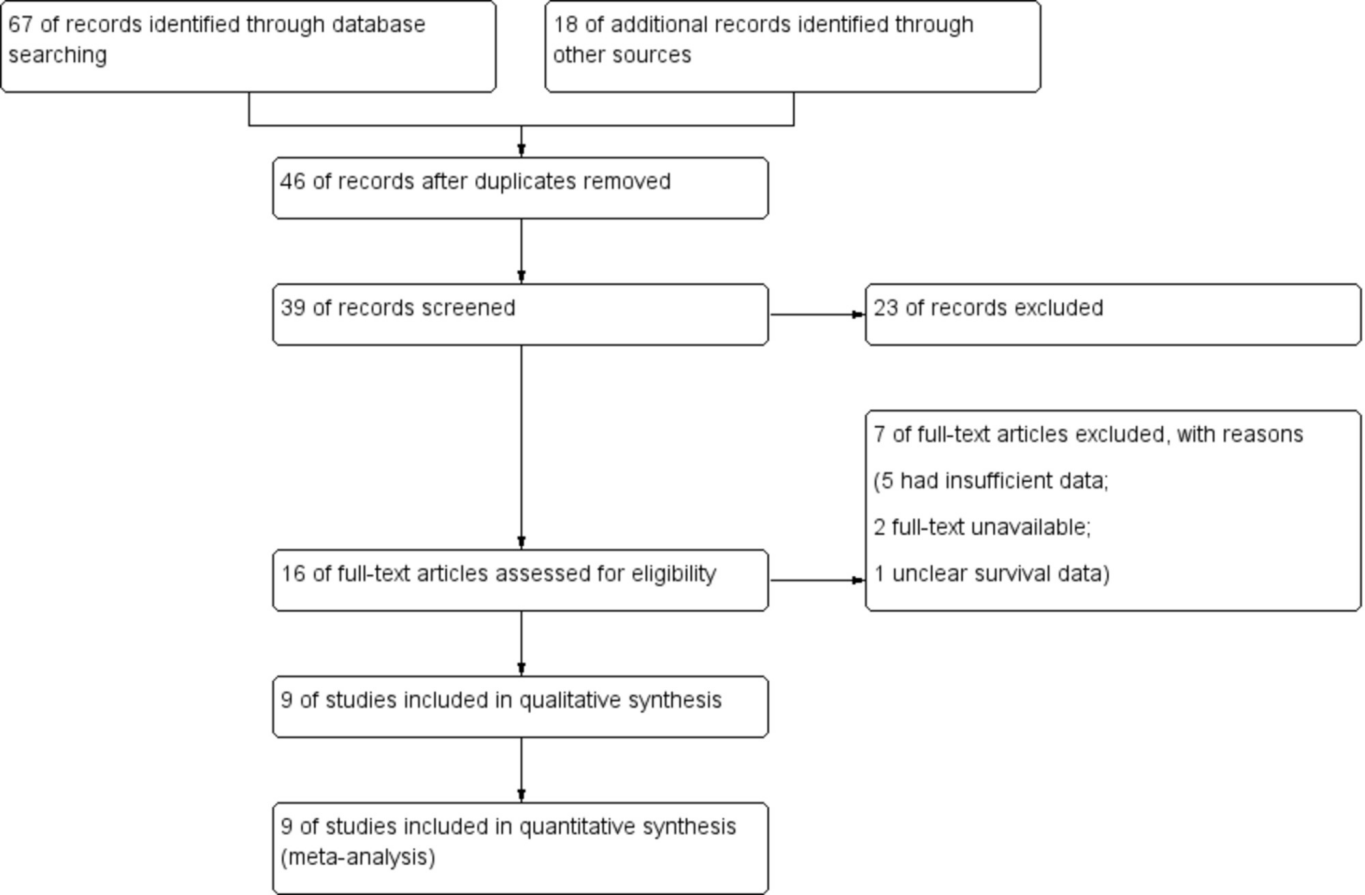
PRISMA flow chart showing study selection PRISMA: Preferred Reporting Items for Systematic Reviews and Meta-Analyses

A total of 85 potentially relevant articles were identified with our predefined search strategy. Based on inclusion and exclusion criteria and following the screening of titles and abstracts, 46 studies were excluded. After excluding duplicates, the reviewers identified 16 studies for an extensive review. Of these, nine studies were entered into the meta-analysis (Figure 1). The quality of articles was assessed by the Oxford Quality Scoring System (OQSS) and was, by and large, acceptable. The main characteristics of the included studies are provided in Table 1.

Meta-analysis results

In the present study, we performed a meta-analysis and examined the association of tumor budding and LNM and OS.

**Figure 2:**
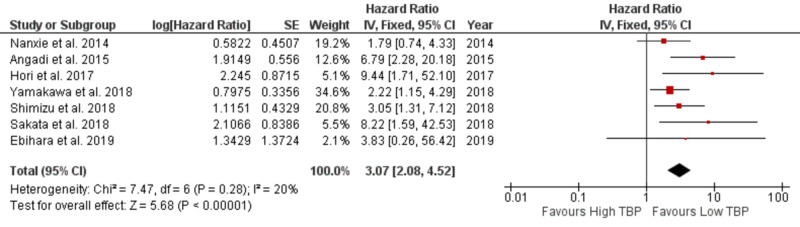
Forest plot showing tumor budding and LNM LNM: Lymph node metastasis; SE: standard error; CI: confidence interval

LNM, as the primary outcome, was extracted from seven studies with available data. A pooled HR and its 95% confidence interval (CI) were calculated with a fixed model for LNM. The pooled HR was 3.07 (95% CI: 2.08-4.52 with I^2^ 20% and P 0.28) for LNM with a statistically significant P <.00001 (Figure 2). The result showed that high tumor budding is associated with LNM.

**Figure 3:**
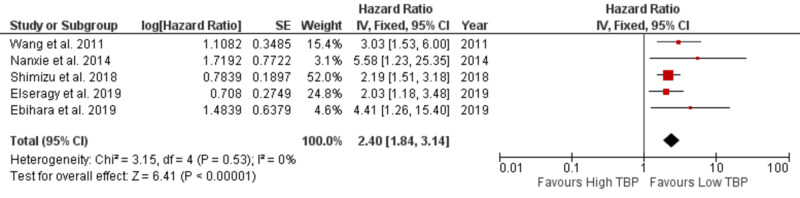
Forest plot showing tumor budding and OS OS: overall survival; SE: standard error; CI: confidence interval

Overall survival, as the primary outcome, was extracted from five studies with available data. The pooled HR was 2.40 (95% CI: 1.84-3.14 with I^2^ 0% and *P* 0.53) for OS with a statistically significant P <.00001 (Figure 3). The result showed that high tumor-budding is associated with poor OS.

Publication bias

**Figure 4:**
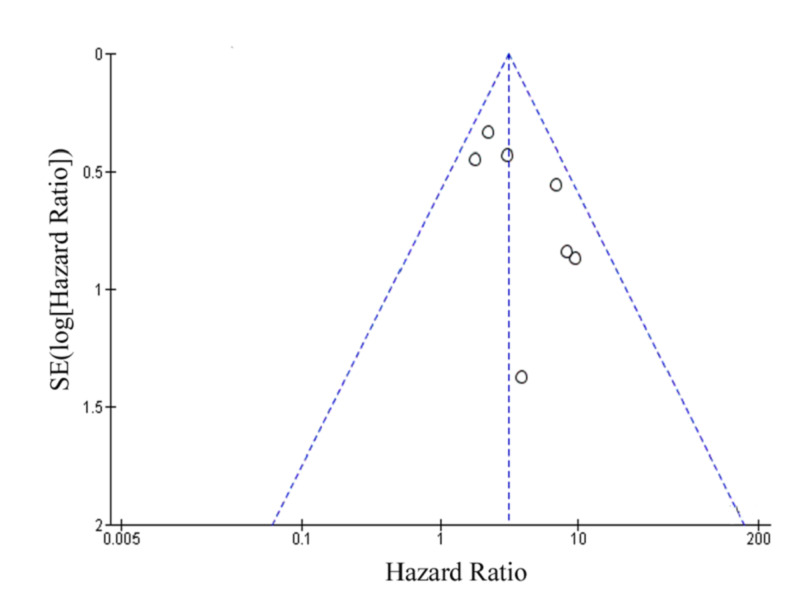
Funnel plot showing tumor budding and LNM LNM: lymph node metastasis; SE: standard error

**Figure 5:**
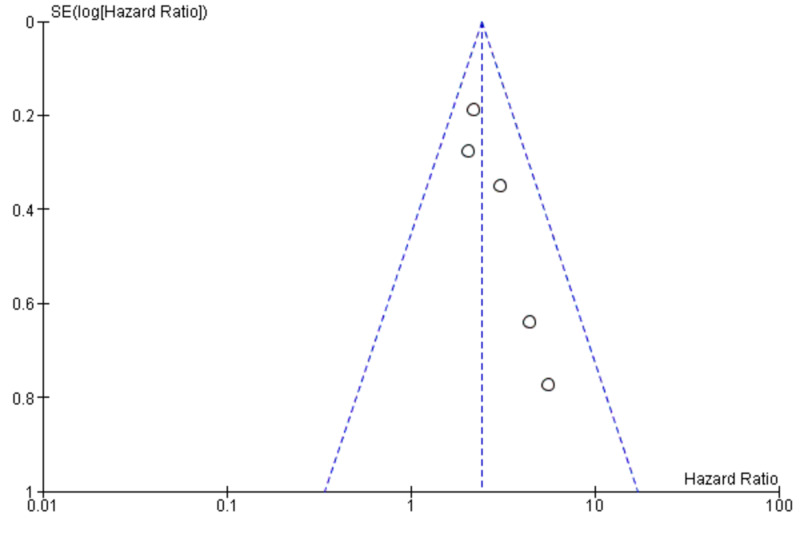
Funnel plot showing tumor budding and OS OS: overall survival; SE: standard error

The publication bias of the included studies was evaluated by funnel plots. No publication bias was established for LNM and OS, as shown in Figure 4 and Figure 5, respectively [3-5,7-12]. This indicated that the publication bias was minute in the current meta-analysis.

## Discussion

Oral squamous cell carcinoma (OSCC) presents with a clinically node-positive disease in about 18%-45% cases [13]. The LNM more commonly seen with OSCC are level I and II cervical lymph nodes. This most common lymph nodal involvement occurs in up to 60%-70% of tongue cancers [14]. The total number of positive nodes has been recognized as a prognostic marker of adverse outcomes [15]. The OS benefit with LNM and the actuarial OS in patients without LNM is 95% and 71%, respectively [16].

Many prognostic markers have been correlated with survival outcome in OSCC, with numerous studies, which repeatedly demonstrated tumor budding as one such promising prognostic marker. In accordance with the International Tumor Budding Consensus Conference (ITBCC 2016) recommendation, tumor budding or sprouting is a continuous phenomenon characterized by the presence of single cancer cells or small clusters of less than five cancer cells outside the main part of the tumor [17-18]. Xie et al. reported an excellent reproducibility and reliability of the ITBCC 2016 scoring method of tumor budding in oral tongue cancer [19]. It is an expression of loss of cellular cohesion and active invasive movement indicating the aggressiveness of the malignancy. It is considered the first histopathological event and initiator of metastasis [20].

This meta-analysis was intended to emphasize the association between tumor budding and LNM and OS in tongue cancer. Our study results demonstrated that a higher tumor budding pattern is associated with an increased incidence of LNM and OS. These findings were further supported by a recent meta-analysis by Almangush et al., which included 16 studies that evaluated the prognostic value of tumor budding in OSCC. They showed that there was a significant association between tumor budding and LNM (odds ratio=7.08, 95% CI=1.75-28.73) and overall OS (HR=1.88, 95% CI=1.25-2.82), adding strength to our results [21].

There are certain limitations in our study that need to be specified. First, there was an inclusion of retrospective studies; hence, there exists a possibility of unavoidable selection bias. Second, there are no randomized controlled trials on this topic that needs to be addressed. Lastly, the absence of prospective studies was also to be noted.

However, the strengths of this meta-analysis are the precision of estimates that are based on a large dataset. This meta-analysis included nine studies involving 1448 patients. The statistical power is satisfactory enough to hold up the results. To our knowledge, this study is the largest analysis of the impact of tumor budding on LNM and OS in oral tongue cancer. The other strengths of this meta-analysis are the precision of tumor budding-specific estimates and the investigation of many covariates. The cut-off value of tumor buds for predicting LNM in each included study is different altogether. The most reliable cut-off value for defining the tumor budding pattern, which could predict the prognosis of tongue cancer patients, is a subject of debate. However, in our study, there is significant statistical power with little heterogeneity when a cut-off of five buds or more is associated with a higher chance of metastasis and poor OS. The other strength of our meta-analysis is the minimal heterogeneity between studies and their subgroups enhances the robustness of the results.

Our findings should be expounded within the order of the effectiveness and limitations of a study-level meta-analysis of heterogeneous studies. Therefore, a large cohort study or an individual patient data meta-analysis is required to assert our results and ascertain the inconsiderable differences.

## Conclusions

Our meta-analysis reviewed the current research targeting the prognostic role of tumor budding in predicting lymph node metastasis and assessing survival in tongue cancer patients. Our findings have demonstrated that a higher tumor budding score is a predictor of poor survival and a higher chance of lymph node metastasis. We conclude that the tumor budding could provide answers for the lacunae in the current TNM staging system.
